# Psychosocial Interventions for Perinatal Common Mental Disorders Delivered by Providers Who Are Not Mental Health Specialists in Low- and Middle-Income Countries: A Systematic Review and Meta-Analysis

**DOI:** 10.1371/journal.pmed.1001541

**Published:** 2013-10-29

**Authors:** Kelly Clarke, Michael King, Audrey Prost

**Affiliations:** 1 UCL Institute for Global Health, University College London, London, United Kingdom; 2 Mental Health Sciences Unit, University College London, London, United Kingdom; Stellenbosch University, South Africa

## Abstract

**Background:**

Perinatal common mental disorders (PCMDs) are a major cause of disability among women. Psychosocial interventions are one approach to reduce the burden of PCMDs. Working with care providers who are not mental health specialists, in the community or in antenatal health care facilities, can expand access to these interventions in low-resource settings. We assessed effects of such interventions compared to usual perinatal care, as well as effects of interventions based on intervention type, delivery method, and timing.

**Methods and Findings:**

We conducted a systematic review, meta-analysis, and meta-regression. We searched databases including Embase and the Global Health Library (up to 7 July 2013) for randomized and non-randomized trials of psychosocial interventions delivered by non-specialist mental health care providers in community settings and antenatal health care facilities in low- and middle-income countries. We pooled outcomes from ten trials for 18,738 participants. Interventions led to an overall reduction in PCMDs compared to usual care when using continuous data for PCMD symptomatology (effect size [ES] −0.34; 95% CI −0.53, −0.16) and binary categorizations for presence or absence of PCMDs (odds ratio 0.59; 95% CI 0.26, 0.92). We found a significantly larger ES for psychological interventions (three studies; ES −0.46; 95% CI −0.58, −0.33) than for health promotion interventions (seven studies; ES −0.15; 95% CI −0.27, −0.02). Both individual (five studies; ES −0.18; 95% CI −0.34, −0.01) and group (three studies; ES −0.48; 95% CI −0.85, −0.11) interventions were effective compared to usual care, though delivery method was not associated with ES (meta-regression β coefficient −0.11; 95% CI −0.36, 0.14). Combined group and individual interventions (based on two studies) had no benefit compared to usual care, nor did interventions restricted to pregnancy (three studies). Intervention timing was not associated with ES (β 0.16; 95% CI −0.16, 0.49). The small number of trials and heterogeneity of interventions limit our findings.

**Conclusions:**

Psychosocial interventions delivered by non-specialists are beneficial for PCMDs, especially psychological interventions. Research is needed on interventions in low-income countries, treatment versus preventive approaches, and cost-effectiveness.

*Please see later in the article for the Editors' Summary*

## Abstract 2

In a systematic review and meta-analysis, Kelly Clarke and colleagues examine the effect of psychosocial interventions delivered by non–mental health specialists for perinatal common mental disorders in low- and middle-income countries.

*Please see later in the article for the Editors' Summary*

## Editors' Summary

**Background:**

Perinatal common mental health disorders are among the most common health problems in pregnancy and the postpartum period. In low- and middle-income countries, about 16% of women during pregnancy and about 20% of women in the postpartum period will suffer from a perinatal common mental health disorder. These disorders, including depression and anxiety, are a major cause of disability in women and have been linked to young children under their care being underweight and stunted.

**Why Was This Study Done?:**

While research shows that both pharmacological (e.g., antidepressants or anti-anxiety medications) and non-pharmacological (e.g., psychotherapy, education, or health promotion) interventions are effective for preventing and treating perinatal common mental disorders, most of this research took place in high-income countries. These findings may not be applicable in low-resource settings, where there is limited access to mental health care providers such as psychiatrists and psychologists, and to medications. Thus, non-pharmacological interventions delivered by providers who are not mental health specialists may be important as ways to treat perinatal common mental health disorders in these types of settings. In this study the researchers systematically reviewed research estimating the effectiveness of non-pharmacological interventions for perinatal common mental disorders that were delivered by providers who were not mental health specialists (including health workers, lay persons, and doctors or midwives) in low- and middle-income countries. The researchers also used meta-analysis and meta-regression—statistical methods that are used to combine the results from multiple studies—to estimate the relative effects of these interventions on mental health symptoms.

**What Did the Researchers Do and Find?:**

The researchers searched multiple databases using key search terms to identify randomized and non-randomized clinical trials. Using specific criteria, the researchers retrieved and assessed 37 full papers, of which 11 met the criteria for their systematic review. Seven of these studies were from upper middle-income countries (China, South Africa, Columbia, Mexico, Argentina, Cuba, and Brazil), and four trials were from the lower middle-income countries of Pakistan and India, but there were no trials from low-income countries. The researchers assessed the quality of the selected studies, and one study was excluded from meta-analysis because of poor quality.

Combining results from the ten remaining studies, the researchers found that compared to usual perinatal care (which in most cases included no mental health care), interventions delivered by a providers who were not mental health specialists were associated with an overall reduction in mental health symptoms and the likelihood of being diagnosed with a mental health disorder. The researchers then performed additional analyses to assess relative effects by intervention type, timing, and delivery mode. They observed that both psychological interventions, such as psychotherapy and cognitive behavioral therapy, and health promotion interventions that were less focused on mental health led to significant improvement in mental health symptoms, but psychological interventions were associated with greater effects than health promotion interventions. Interventions delivered both during pregnancy and postnatally were associated with significant benefits when compared to usual care; however, when interventions were delivered during pregnancy only, the benefits were not significantly greater than usual care. When investigating mode of delivery, the researchers observed that both group and individual interventions were associated with improvements in symptoms.

**What Do These Findings Mean?:**

These findings indicate that non-pharmacological interventions delivered by providers who are not mental health specialists could be useful for reducing symptoms of perinatal mental health disorders in middle-income countries. However, these findings should be interpreted with caution given that they are based on a small number of studies with a large amount of variation in the study designs, settings, timing, personnel, duration, and whether the intervention was delivered to a group, individually, or both. Furthermore, when the researchers excluded studies of the lowest quality, the observed benefits of these interventions were smaller, indicating that this analysis may overestimate the true effect of interventions. Nevertheless, the findings do provide support for the use of non-pharmacological interventions, delivered by non-specialists, for perinatal mental health disorders. Further studies should be undertaken in low-income countries.

**Additional Information:**

Please access these websites via the online version of this summary at http://dx.doi.org/10.1371/journal.pmed.1001541

- The World Health Organization provides information about perinatal mental health disorders
- The UK Royal College of Psychiatrists has information for professionals and patients about perinatal mental health disorders

## Introduction

Common mental disorders, defined as depressive, anxiety, and somatic disorders, are a major cause of disability among women during the perinatal period, and may have consequences for children's growth and development [1]–[4]. In low- and lower middle-income countries an estimated 16% (95% CI 15.0%, 16.8%) of women suffer from these disorders in pregnancy, and around 20% (95% CI 19.2%, 20.6%) in the postnatal period [5]. To date, most reviews of interventions for perinatal common mental disorders (PCMDs) have focused on interventions for depression, and on evidence from high-income countries [6]–[10]. Their results may not be generalizable to low-resource settings, where specialists and financial resources for mental health care are scarce [11]–[13]. In these settings, the World Health Organization Mental Health Gap Action Programme recommends a cost-effective package of interventions to treat depression that includes antidepressant, psychoeducation, and problem-solving therapies [14]. A recent meta-analysis showed that interventions for PCMDs in low- and middle-income countries are effective (effect Size [ES] −0.38; 95% CI −0.56, −0.21), with benefits for children's health and cognitive development, and for the quality of mother–infant interactions [15]. The findings from this review, though useful, are limited by the diversity of interventions included and high statistical heterogeneity (*I* ^2^ = 79.9%). Effects of different intervention types and statistical heterogeneity were not fully investigated.

We have conducted a systematic review and meta-analysis of interventions for PCMDs in low- and middle-income countries that address the limitations of previous reviews. We include interventions for all PCMDs since depression and anxiety often coexist, and subcategories of common mental disorder may lack conceptual validity in some cultures [16]–[18]. We focus on psychosocial interventions (i.e., non-pharmacological interventions to influence thoughts, behaviors, skills, and associated feelings), given concerns about the safety of pharmacotherapy during the perinatal period and because access to psychotropic drugs and trained personnel to prescribe them can be limited in low-resource settings [19]–[22]. We also focus on interventions delivered by providers without specialized mental health training (“non-mental health specialists”) in community and primary care settings because of the lack of mental health professionals in low- and middle-income countries, and to address calls for integration of mental health interventions into existing community and maternal and child health programs [23],[24]. We investigate the effects of these interventions based on the type of intervention, timing, and delivery mode, in order to make practical policy recommendations.

## Methods

We conducted the systematic review in accordance with the 2009 PRISMA (Preferred Reporting Items for Systematic Reviews and Meta-Analyses) statement (Text S1) [25]. The protocol was finalized prior to conducting the systematic review and meta-analysis (Protocol S1). The review was not registered with PROSPERO or any other database.

### Criteria for Trials Considered for the Review

#### Study types and origins

We considered published and unpublished, randomized and non-randomized controlled trials. Publication dates were not restricted, but only trials written in English, French, or Spanish were included. We restricted the review to trials conducted in low- and middle-income countries according to World Bank country classifications at the time of the search [26]. We included studies from mainland China because it is a middle-income country. However, we excluded studies from Taiwan and Hong Kong Special Administrative Region because economic conditions and health infrastructure in these regions of China are comparable with those of high-income settings.

#### Participants

We included trials that enrolled pregnant or postnatal women (≤12 mo after delivery), or women who were not pregnant at recruitment but became pregnant during the trial.

#### Interventions

We considered preventive and treatment interventions involving a psychological or social component, delivered prior to pregnancy, during pregnancy, and/or postnatally. We included interventions delivered by non-mental health specialists, including lay persons (i.e., those without any health training), health workers and health volunteers (with some health training), and nurses and doctors with no specialized mental health training. We excluded interventions delivered by psychiatrists, psychologists (undergraduate or postgraduate level), and psychosocial workers, as these practitioners are not commonly available in low- and middle-income settings. We considered interventions in community settings (e.g., villages) and, knowing that the antenatal period is the time when most women are likely to come into contact with health services, the most commonly accessible health provider of antenatal care for their location (e.g., health posts, primary care centers, and hospitals).

#### Types of outcome measures

We included antenatal and postnatal PCMDs since a large proportion of PCMDs identified in the postnatal period are also present during pregnancy [27]–[29]. There is no consistent definition of the perinatal period in the psychiatric literature, so we adopted a working definition of pregnancy plus the first 12 mo after birth, in line with a number of trials [30]–[32]. We included trials measuring depressive, anxiety, panic, and somatic disorders, as well as perinatal psychological distress as a proxy measure of PCMDs [1]. We considered trials where outcomes were defined and measured using structured clinical interviews, such as the Clinical Interview Schedule–Revised [33], or validated screening questionnaires, for example, the Edinburgh Postnatal Depression Scale [34], the Kessler 10-Item Scale [35], and the 12-item General Health Questionnaire [36]. Outcomes included in the review were binary categorizations indicating the presence or absence of a PCMD (“caseness”), and reduction of symptoms of PCMDs as a continuous outcome.

### Search Methods for Identifying Trials

Between 5 and 7 July 2013 we searched the following online bibliographic databases for trials that met the inclusion criteria detailed above: Medline, Embase, Cumulative Index to Nursing and Allied Health Literature, the Cochrane Library, PsycINFO, Web of Science, Scopus, Popline, Maternity and Infant Care, and the Global Health Library. We searched for unpublished completed or ongoing trials in the World Health Organization International Clinical Trials Registry. Customized search strategies were developed for each database. We used controlled vocabulary (e.g., MeSH terms) and search filters to identify randomized controlled trials, and trials from low- and middle-income countries where these were available. Our search of Embase (via OVID), including the exact search terms, is included in Text S2. The search mainly identified journal articles, but also reports, conference proceedings, and theses. We contacted experts in the field to identify further relevant trials, specifically unpublished or ongoing trials.

### Data Collection and Analysis

#### Trial selection and data extraction

We removed duplicates and articles not written in English, French, or Spanish, and reviewed the abstracts of the remaining articles. Trials of interventions were retained, and observational studies excluded. We searched reviews of appropriate interventions for relevant citations. We contacted authors to request full articles where they were not available online, as well as further details of interventions as required. One reviewer (K. C.) independently screened full articles that appeared to meet the search criteria to assess the trial setting and design. We resolved any uncertainty about the inclusion of specific trials through discussions between reviewers, and documented reasons for exclusion.

Using a spreadsheet, two reviewers (K. C. and A. P.) independently recorded the following data for included trials: date of extraction, source reference and type, authors, publication year, article title, source of funding, trial design and methods, study setting and population, details about interventions and control conditions, participant inclusion and exclusion criteria and characteristics, sample size, definitions of PCMDs, screening tools, timing of assessments, variables adjusted for in the analyses, and results.

#### Assessment of methodological quality and small study effects

We did not exclude papers from the systematic review on the basis of methodological quality but assessed risk of bias for each study in terms of sequence generation, allocation concealment, blinding, incomplete outcome data, and selective reporting, using the Cochrane Risk of Bias Tool [37]. We defined trials at high risk of bias as those found to be at high risk or unclear risk of bias across five or more bias domains. Trials at low risk of bias were defined as those using adequate sequence generation and allocation concealment methods [37]. In reality these definitions were arbitrary, and studies may lie anywhere along the continuum from “free of bias” to “undoubtedly biased” [38]. We assessed potential small study effects using a funnel plot and the Egger test [38]. However, we attempted to limit small study effects by searching the World Health Organization International Clinical Trials Registry, and by asking expert informants about unpublished and ongoing trials.

#### Data synthesis and statistical analysis

We identified more than six studies that were not at high risk of bias and were comparable in terms of intervention content and study population [39]. We therefore conducted a meta-analysis to assess effects of psychosocial interventions versus usual care. We used the main outcomes reported in each publication, adjusted for clustering, baseline differences, and other covariates where appropriate. We conducted separate meta-analyses for binary and continuous outcomes. Odds ratios (ORs) were pooled for trials reporting binary outcomes. Where studies reported binary outcomes from both clinical interviews and screening questionnaires, we selected the former as the superior measure of PCMDs. One study reported a categorical outcome for the presence of PCMD (none/mild, moderate, or severe) [40]. Data were therefore reanalyzed to calculate a binary outcome (none/mild versus moderate/severe) using the same methods reported in the publication. For continuous outcomes, standardized mean differences were calculated because different screening questionnaires were used to report the outcome [38]. We estimated statistical heterogeneity using the *I* ^2^ statistic and calculated confidence intervals around these estimates [41],[42]. We used a random effects model to account for unexplained heterogeneity and because we assumed that the effects being estimated in the different trials were not identical [38]. We planned to exclude trials at high risk of bias from the meta-analysis (although all trials were included in the systematic review), and to conduct one sensitivity analysis including only trials at low risk of bias [38] and another including only results from the last follow-up assessment in each trial. We conducted a further post hoc sensitivity analysis excluding a study that was not peer-reviewed.

We planned to conduct the following subgroup analyses: psychological interventions versus usual care, health promotion interventions versus usual care, group-based interventions versus usual care, individual-based interventions versus usual care, and combined (group- and individual-based) interventions versus usual care. We carried out two further post hoc subgroup analyses: antenatal interventions versus usual care, and antenatal and postnatal interventions versus usual care.

We wanted to compare treatment and preventive approaches versus usual care, but this was not possible because only one of the retrieved studies was a trial of a treatment intervention. In order to maximize power for subgroup analyses, we pooled results from all trials by converting ORs to ESs—comparable with the standardized mean difference—where studies did not report a continuous outcome [43]. In order to examine differences in ES between intervention subgroups we conducted a series of univariable random effects meta-regression analyses [44].

All data analyses were conducted with Stata (version 12.1) using metan and metareg commands.

## Results

**Figure 1:**
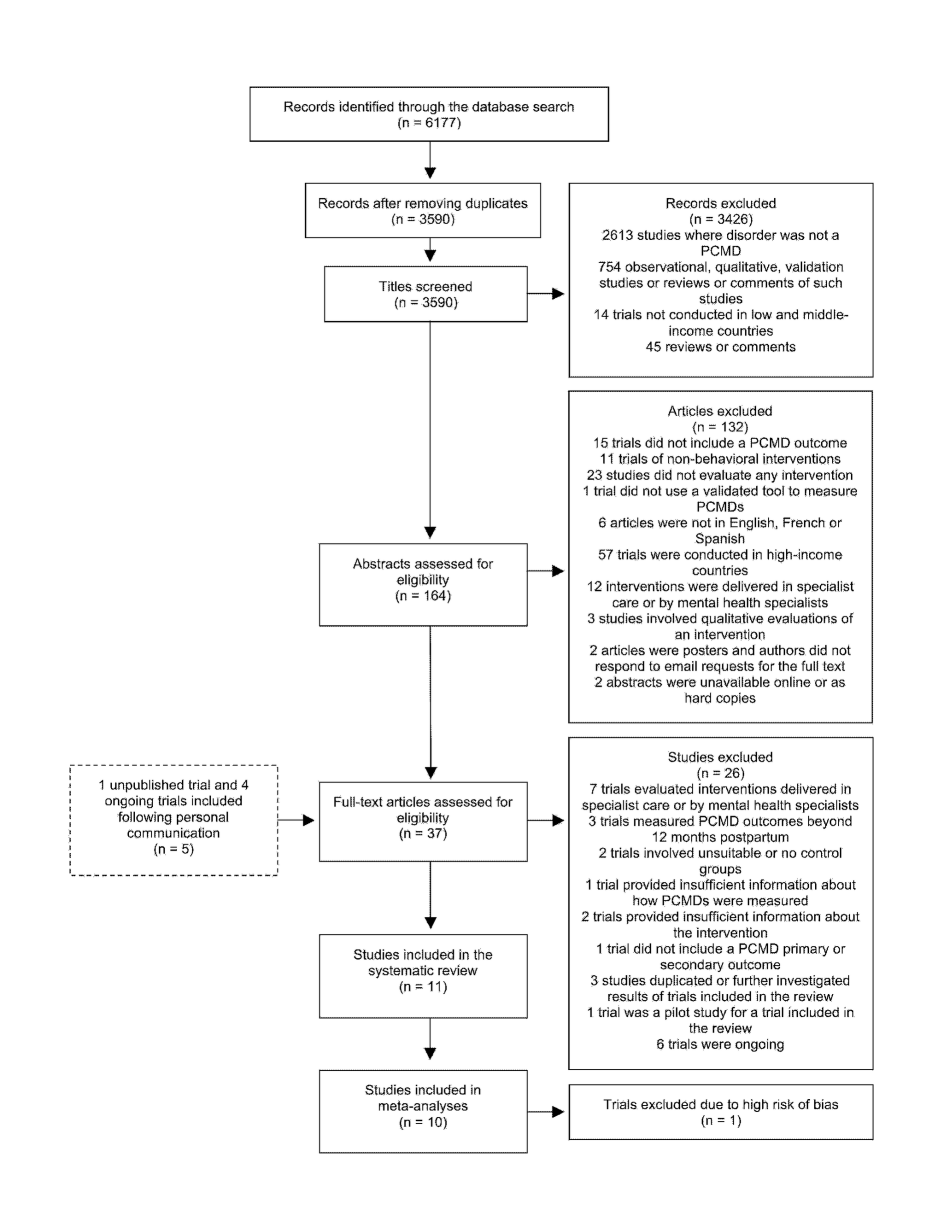
Flow diagram of search results. Out of 6,177 abstracts retrieved through a search of electronic databases, 11 articles were included in the systematic review, including one unpublished trial identified following personal communication with the author.

The database search identified 6,177 abstracts, which we screened according to the process outlined in Figure 1. We also identified five trials through personal communication with researchers. Two abstracts were unavailable online through University of London or British Library accounts. We were unable to obtain these abstracts through colleagues working in Asian institutions and could not locate the authors' e-mail addresses to contact them directly [45],[46]. We screened 37 full-text articles, and trials excluded at this stage are shown in Table S1, with reasons for exclusion. Six ongoing trials, including one conducted in a low-income country, were not included in the review but are described in Table S2. In total, 11 trials were included in the review and are described in Table 1. Results from one trial are reported in two separate publications [47],[48].

**Table 1:** Components of psychosocial interventions for PCMDs delivered by non-mental health specialists in middle-income countries.

Study	Setting	Intervention	Control Group	Delivery Mode	Personnel	Timing of Intervention	Duration and Number of Sessions	Sessions per Month	Target Population	Target Disorder	Timing of Assessment/s	Number of Participants			Number of Participants Lost to Follow-Up (in Final Analyses)		
												Con	Int	Tot	Con	Int	Tot
**Treatment interventions**																	
Rahman 2008 [56]	Community	Home-based intervention using CBT techniques	Enhanced care involving home visits by health workers	Individual	Community health workers	Third trimester of pregnancy and 10 mo postnatally	16 individual sessions over 11 mo	1.5	Pregnant married women aged 16–45 y, married, with depression	Antenatal and postnatal depression	6 and 12 mo postnatally	440	463	903	54	51	105
**Preventive interventions**																	
Cooper 2009 [52]	Community	Home visits to encourage sensitive responsive interactions between mother and infant	Usual care	Individual	Lay women	Antenatal and up to 6 mo postnatally	16 individual sessions over 6+ mo	Average of 2.3	Pregnant women residing in the project area	Postnatal depression	6 and 12 mo postnatally	229	220	449	52	55	107
Futterman 2010 [51]	Health clinics	Group CBT sessions conducted by mentor mothers; individual sessions with mentor mothers	Usual care	Groups and individual	Peer mothers	Antenatal and postnatal for individual sessions; not reported for group sessions	8 group sessions; number of individual sessions unclear	Not reported	Pregnant women attending study clinics, diagnosed as HIV-positive	Postnatal depression	6 mo postnatally	77	83	160	?	?	89
Gao 2010 [47] and Gao 2012 [48]	Hospital	Antenatal ITP program and postnatal follow-up call	Usual care	Groups	Midwives	Antenatal and postnatal	2 antenatal group sessions and a phone call within 2 wk after delivery	0.75	Nulliparous pregnant women aged 35 y or younger, married, and living with husbands	Postnatal depression	6 wk and 3 mo postnatally	98	96	194	10	9	19
Hughes 2008 [57]	Community	Home visits delivered by an experienced mother; sessions used active listening and were centered on the mother	Usual care plus postnatal visit for assessment of mental health	Individual	Lay women (experienced mothers)	Antenatal and postnatal	2 group sessions and 3 individual sessions over 14 wk	1.6	Pregnant women at risk of developing postnatal depression	Postnatal depression	12 and 26 wk postnatally	210	212	422	29	25	54
Langer 1996 [54]	Antenatal clinics	Home visits centered on improving social support, knowledge about perinatal health, and health care	Usual care	Individual	Female social workers or obstetric nurses	Antenatal	4–6 individual sessions over 12–14 wk	1.2–2.2	Pregnant women with one or more risk factors for stress during pregnancy	Antenatal/postnatal anxiety	36th week of pregnancy and 40 d postnatally	1,125	1,110	2,235	162	146	308
Le Roux 2013 [50]	Community	A home-visiting intervention involving an average of six antenatal and five postnatal home visits focused on maternal health and nutrition, breastfeeding, antenatal health care, HIV testing, and stopping alcohol use, as well as issues related to child health, including immunization, prevention of HIV transmission, and infant bonding	Usual care including HIV care at government clinics and hospitals	Individual	Community health workers	Antenatal and postnatal	On average, 11 visits over a maximum of 5 mo (3 mo antenatally and 2 mo after childbirth)	2.2	Pregnant women aged 18 y or older, living in the study neighborhoods	Postnatal depression	6 mo postnatally	594	644	1,238	37	71	108
Mao 2012 [49]	Hospital	Emotional self-management group training program based on CBT, involving group sessions and one counseling visit at home	Usual care	Groups and individual	Obstetricians	Antenatal	4 group sessions and 1 individual session over approximately 4 wk	5.0	Primiparous women attending study clinic, without pregnancy complications or a family or personal history of mental illness	Antenatal/postnatal depression	36th week of pregnancy and 6 wk postnatally	120	120	240	12	7	19
Rahman 2009 [55]	Community	Workshops and home visits centered on infant development	Usual care	Groups and individual	Community health workers	Postnatal	Group workshop plus fortnightly home visits	Not reported	Pregnant women aged 17–40 y residing in intervention clusters	Postnatal distress	12 wk postnatally	173	194	367	27	31	58
Robledo-Colonia 2012 [53]	Primary care	An antenatal aerobic exercise program, involving three 60- min exercise classes per week, starting between week 16 and 20 of gestation and continuing for 3 mo	Usual care	Groups	Physiotherapist	Antenatal	3 group sessions per week for 3 mo	12.0	Pregnant women aged 16–30 y, without current or a history of chronic medical illness including mental illness	Antenatal depression	28th–32nd week of pregnancy	40	40	80	3	3	6
Tripathy 2010 [40]	Community	Women's groups working through participatory learning and action cycle to address maternal and child health problems	Usual care plus formation of cluster-level health committees and workshops for appreciative enquiry with government health staff	Groups	Lay women	All women residing in the study area were welcome to join a group; pregnant women were preferentially invited	20 group sessions over 20 mo	1.0	Postnatal women residing in the intervention clusters	Postnatal distress	6 wk postnatally	6,097	6,513	12,429	118	63	181

?, not reported; Con, control arm; Int, intervention arm; Tot, total.

### Included Trials

None of the trials included were conducted in a low-income country. Seven of the included trials were conducted in upper middle-income countries: China [47],[49], South Africa [50]–[52], Columbia [53], Mexico, Argentina, Cuba and Brazil [54]. Four trials were set in lower middle-income countries: Pakistan [55],[56] and India [40],[57].

### Trial Characteristics

#### Outcomes

Depression was an outcome in eight trials [47],[49]–[53],[56],[57], and anxiety in only one trial [54]. Three trials measured general common mental disorders [40],[47],[55]. All trials employed validated self-report measures to assess PCMD symptomatology: five used the Edinburgh Postnatal Depression Scale [47],[49],[50],[52],[57], others used self-report measures such as the Center for Epidemiological Studies Depression Scale [51],[53], the Kessler 10-Item Scale [40], the Self Reporting Questionnaire [55], the Hamilton Depression Rating Scale [56], the nine-item Patient Health Questionnaire [49], and the 12-item General Health Questionnaire [47]. Only four trials used a clinical interview in addition to self-report measures [49],[52],[56],[57].

#### Target populations

Table 1 details components of interventions included in the review. We defined treatment interventions as those that targeted women diagnosed with a PCMD, and preventive interventions as those sampling from the general population, women at risk of developing a PCMD, and women with symptoms but not meeting the full criteria for a PCMD [58]. We identified only one treatment intervention [56].

In ten trials, participants were recruited during pregnancy [47],[49]–[57]. In Tripathy et al., all mothers who had delivered in the study area were interviewed approximately 6 wk after childbirth [40]. The intervention (participatory women's groups) was delivered at a community level: 18% of participants attended groups in the first year, rising to 55% in the third year. Overall for the included studies, participants' initial exposure to the intervention may therefore have occurred prior to or during pregnancy, in the postnatal period, or not at all.

#### Interventions

Six trials involved community-based interventions, three of which were conducted in resource-limited, rural settings [40],[50],[52],[55]–[57]. Five trials involved interventions based in health facilities, including primary care facilities [53], antenatal clinics [51],[54], and hospitals [47],[49]. Four out of five facility-based interventions were conducted in urban populations.

The timing of interventions varied: three trials tested interventions limited to pregnancy [49],[53],[54], and one to the postnatal period [55]. In six trials, interventions began antenatally and continued into the postnatal period [40],[47],[50],[52],[56],[57]. One trial did not report the timing of the intervention [51].

The duration of interventions ranged from 4 wk [49] to 20 mo [40]. Where appropriate, we compared their intensity by calculating the number of scheduled contact events (group or individual) per month (Table 1). Of six trials, the least intensive intervention involved two group sessions plus a follow-up telephone call over a period of 9.5 mo [47]. The most intensive intervention involved three group exercise sessions per week for 3 mo [53].

### Intervention Content

The treatment intervention and three of the ten preventive interventions involved psychological components [47],[49],[51],[56]. Psychological interventions were defined as interventions incorporating a structured and explicitly psychological approach, such as cognitive behavior therapy (CBT) or interpersonal therapy (IPT).

Seven trials tested health promotion interventions [40],[50],[52]–[55],[57]. Health promotion approaches were defined by the absence of a structured and explicitly psychological approach, and incorporation of one of the following components: communication techniques to positively influence individuals and communities; education to improve knowledge and skills conducive to health; sharing of common experiences or problems and social support; creation of better environments to promote healthier living; community development and mobilization to address health problems; or advocacy and health policy development [59].

Two health promotion interventions involved educational workshops and/or home visits, specifically focusing on mother–infant interactions and attachment [52],[55]. One intervention was a participatory learning and action cycle to improve maternal and newborn health, through women's groups [40]. Groups were also used to deliver an antenatal exercise program incorporating motivating techniques, including support by a physiotherapist, exercise with other women, and music [53]. Two interventions used home visits to communicate information to participants about topics including perinatal health care, nutrition, and mother–infant interaction [50],[57]. One of these interventions promoted infant gender equality and had a strong emphasis on listening to participants [57]. Home visits were used in another intervention to disseminate information about pregnancy and delivery to participants and their chosen “support persons” [54].

Details of care received by control groups are included in Table 1.

### Intervention Delivery Mode and Personnel

Psychological interventions were delivered by health workers [56], lay persons (“mentor mothers” [51]), and doctors or midwives [47],[49]. Three out of four psychological interventions were predominantly delivered in a group context [47],[49],[51]; one psychological intervention was delivered during individual home visits [56].

Health promotion interventions were delivered to groups and individuals by community health workers, social workers, physiotherapists, obstetric nurses, and lay women.

### Methodological Quality of Trials and Risk of Bias

All but one of the studies had been peer-reviewed [57]. Most used self-report measures validated in the study population, and ten used a measure validated in the country in which they were conducted [40],[47],[49]–[55],[57]. One trial in Pakistan used the Hamilton Depression Rating Scale, which had not been formally validated in this context but which was translated, culturally adapted, and administered by experienced mental health professionals [56]. Statistical analysis in three trials included in the intervention did not take account of clustering [47],[49],[53].

For each trial we assessed risk of bias, as summarized in Table 2. Sequence generation for randomization was adequate in nine trials [40],[47],[49],[50],[52],[54]–[57], unclear in one trial [53], and absent in one non-randomized trial in which participants were allocated by clinic [51]. Method of allocation concealment was adequate in seven trials [40],[50],[52],[54]–[57], unclear in three trials [47],[49],[53], and absent in one [51].

**Table 2:** Assessment of risk of bias for trials included in the review.

Study	Random Sequence Generation	Allocation Concealment	Blinding of Participants and Personnel	Blinding of Outcome Assessment	Complete Outcome Data	No Selective Reporting
Cooper 2009 [52]	✓	✓	✗	✓	✓	?
Futterman 2010 [51]	✗	✗	✗	?	✗	?
Gao 2010 [47],[48]	✓	?	✗	✓	✓	?
Hughes 2008 [57]	✓	✓	✗	✓	✓	✓
Langer 1996 [54]	✓	✓	✗	✓	?	?
Le Roux 2013 [50]	✓	✓	✗	?	✓	✗
Mao 2012 [49]	✓	?	✗	?	✓	?
Rahman 2008 [56]	✓	✓	✓	✓	✓	✓
Rahman 2009 [55]	✓	✓	✗	✓	✓	?
Robledo-Colonia 2012 [53]	?	?	✗	✓	✓	?
Tripathy 2010 [40]	✓	✓	✗	✗	✓	✓

✓ = yes (low risk of bias); ✗ = no (high risk of bias); ? = unclear risk of bias.

The nature of interventions inhibited blinding of participants and personnel in most trials; however, blinding of outcome assessors occurred in seven trials [47],[52]–[57]. Outcome assessments were not blinded in one trial [40], and three provided insufficient details [49]–[51].

With regards to completeness of follow-up data, the information provided was adequate in nine trials [40],[47],[49],[50],[52],[53],[55]–[57] and inadequate in one trial [51]; in another trial, reasons for loss to follow-up were not discussed [54]. Two trials reported high attrition rates: 24% at 12 mo [52] and 55.6% at 6 mo [51]. We were unable to assess selective reporting (defined as the occurrence of one of the following: not all of a study's prespecified outcomes reported, primary outcomes not prespecified, outcomes incompletely reported, or key outcomes expected to be reported not reported) in the majority of trials for which the study protocol was not available.

### Intervention Effects: Meta-Analysis

Out of the 11 trials that met the inclusion criteria, ten had useable outcomes for 18,738 participants [40],[47],[49],[50],[52]–[57]. One trial found to be at high risk of bias (which had not used adequate sequence generation and allocation concealment methods) was excluded from the meta-analysis to reduce the impact of bias on the results [38],[51].

### Comparison 1: All Interventions versus Usual Care

**Figure 2:**
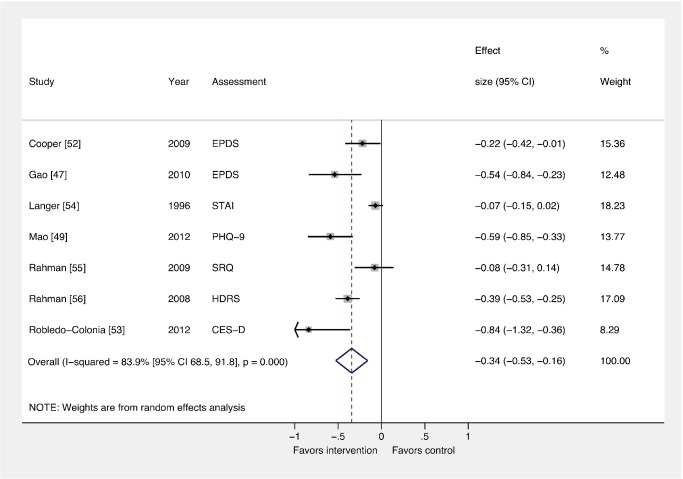
Effects of psychosocial interventions on continuous PCMD outcomes. The pooled effect of interventions delivered by non-mental health specialists compared to usual perinatal care was a reduction in PCMD symptomatology compared to usual care, using effect estimates from assessments immediately following delivery of the intervention (ES −0.34; 95% CI −0.53, −0.16). CES-D, Center for Epidemiological Studies Depression Scale; EPDS, Edinburgh Postnatal Depression Scale; HDRS, Hamilton Depression Rating Scale; PHQ-9, nine-item Patient Health Questionnaire; SRQ, Self Reporting Questionnaire; STAI, State-Trait Anxiety Inventory.

**Figure 3:**
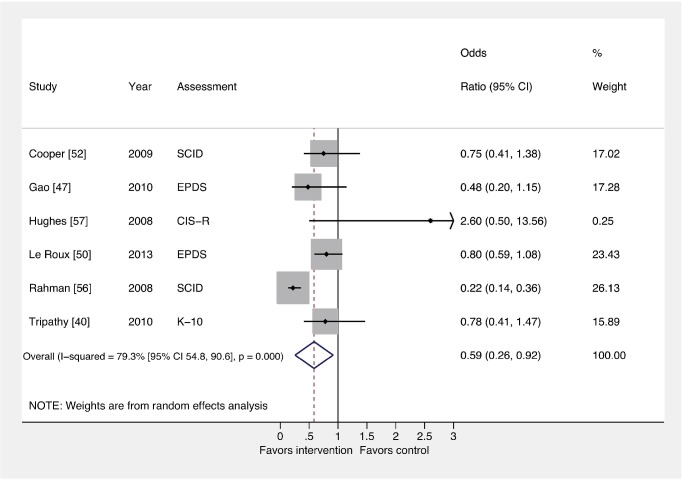
Effects of psychosocial interventions on binary PCMD outcomes. Using binary PCMD categorizations from assessments immediately following delivery of the intervention, the pooled effect for all interventions was significant (OR 0.59; 95% CI 0.26, 0.92) compared to usual care. CIS-R, Clinical Interview Schedule–Revised; EPDS, Edinburgh Postnatal Depression Scale; K-10, Kessler 10-Item Scale; SCID, Structured Clinical Interview for DSM Disorders.

Figures 2 and 3 show the pooled effects of any intervention (ten in total) versus usual care, for dichotomous (Figure 2) and continuous outcomes (Figure 3), after intervention. There was evidence that interventions delivered by non-mental health specialists compared to usual perinatal care were associated with a reduction in PCMD symptoms (ES −0.34; 95% CI −0.53, −0.16) and caseness (OR 0.59; 95% CI 0.26, 0.92) immediately after the intervention. Heterogeneity was high (*I* ^2^ = 84.1% and 79.3%, respectively) and statistically significant.

We conducted sensitivity analyses excluding the study that was not peer-reviewed [57], and using binary and continuous outcomes associated with the final assessment, as opposed to the assessment immediately after the intervention. These analyses resulted in similar ESs. We also performed a sensitivity analysis using studies with low risk of bias and found that the ES was reduced for PCMD symptoms and caseness (ES −0.19; 95% CI −0.36, −0.02; OR 0.61; 95% CI 0.22, 1.01). Statistical heterogeneity was not significantly reduced in any of these sensitivity analyses.

In order to pool results from all ten trials of psychosocial interventions, we converted ORs to ESs where trials did not report a continuous outcome. The pooled ES of converted and unconverted outcomes was significant (ES −0.27; 95% CI −0.42, −0.13). A funnel plot of these outcomes was broadly symmetric (Figure S1), though the power to detect small study effects with this method was low given the small number of trials included in the meta-analysis. The Egger test provided no evidence of small study bias on PCMD symptoms (*p* = 0.205).

### Comparison 2: Interventions by Type versus Usual Care

**Figure 4:**
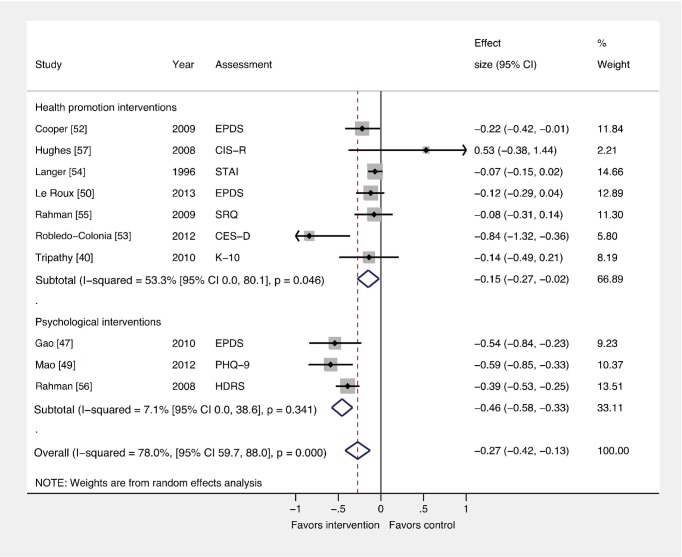
Effects of psychological and health promotion interventions on continuous PCMD outcomes. The pooled effect of three health promotion interventions delivered by non-mental health specialists was significant compared to usual care (ES −0.15; 95% CI −0.27, −0.02). Three psychological interventions were associated with a larger overall ES (−0.46; 95% CI −0.58, −0.33). CES-D, Center for Epidemiological Studies Depression Scale; CIS-R, Clinical Interview Schedule–Revised; EPDS, Edinburgh Postnatal Depression Scale; HDRS, Hamilton Depression Rating Scale; K-10, Kessler 10-Item Scale; PHQ-9, nine-item Patient Health Questionnaire; SCID, Structured Clinical Interview for DSM Disorders; SRQ, Self Reporting Questionnaire; STAI, State-Trait Anxiety Inventory.

We conducted a subgroup analysis to assess whether ESs differed by intervention type (Figure 4). This analysis was important because heterogeneity was high in the main comparison (Comparison 1), and subgroup analyses can provide explanations for heterogeneity. Health promotion interventions for PCMDs were evaluated in seven trials with a total of 17,401 participants, and these interventions were beneficial compared to usual care (ES −0.15; 95% CI −0.27, −0.02). Psychological interventions, evaluated in three trials with a total of 1,337 participants, had a larger effect (ES −0.46; 95% CI −0.58, −0.33). In a meta-regression analysis, the ESs for psychological and health promotion interventions were significantly different (β coefficient −0.33; 95% CI −0.09, −0.58) (Table S3).

### Comparison 3: Interventions by Delivery Method versus Usual Care

**Figure 5:**
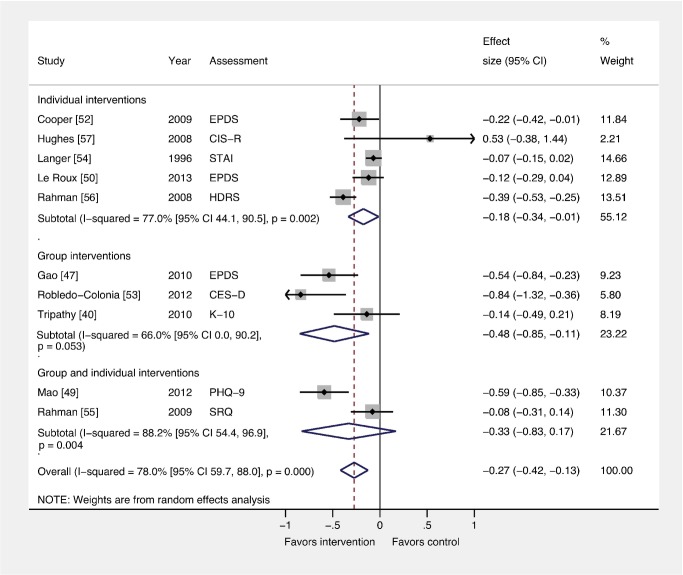
Effects of group and individually based psychosocial interventions on continuous PCMD outcomes. Individual (ES −0.18; 95% CI −0.34, −0.01) and group-based (ES −0.48; 95% CI −0.85, −0.11) psychosocial interventions were associated with significant ESs for PCMDs compared to usual care. Interventions combining group and individual components had no significant effect compared to usual care. CES-D, Center for Epidemiological Studies Depression Scale; CIS-R, Clinical Interview Schedule–Revised; EPDS, Edinburgh Postnatal Depression Scale; HDRS, Hamilton Depression Rating Scale; K-10, Kessler 10-Item Scale; PHQ-9, nine-item Patient Health Questionnaire; SRQ, Self Reporting Questionnaire; STAI, State-Trait Anxiety Inventory.

We also conducted subgroup analyses to examine whether ESs differed by intervention delivery method (Figure 5). Five trials (*n* = 5,247) and three trials (*n* = 12,884) evaluated individual and group-based interventions, respectively. Although individual (ES −0.18; 95% CI −0.34, −0.01) and group (ES −0.48; 95% CI −0.85, −0.11) interventions for PCMDs were effective compared to usual care, delivery method was not associated with ES (β −0.11; 95% CI −0.36, 0.14). Interventions with combined group and individual components had no benefits compared to usual care (ES −0.33; 95% CI −0.83, 0.17).

### Comparison 4: Interventions by Timing versus Usual Care

**Figure 6:**
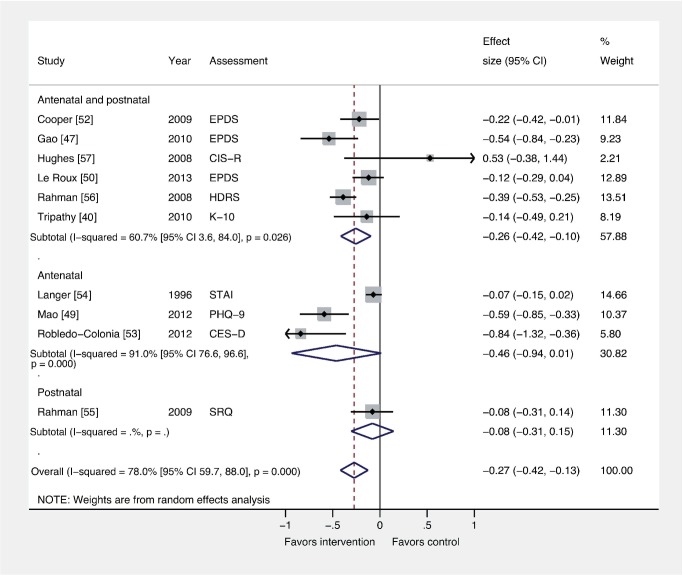
Effects of antenatal and postnatal psychosocial interventions on continuous PCMD outcomes. Antenatal interventions were not effective for PCMDs compared to usual care (ES −0.46; 95% CI −0.94, 0.01), whereas interventions delivered both antenatally and postnatally were (ES −0.26; 95% CI −0.42, −0.10). Only one trial assessed an intervention delivered in the postnatal period only. CES-D, Center for Epidemiological Studies Depression Scale; CIS-R, Clinical Interview Schedule–Revised; EPDS, Edinburgh Postnatal Depression Scale; HDRS, Hamilton Depression Rating Scale; K-10, Kessler 10-Item Scale; PHQ-9, nine-item Patient Health Questionnaire; SRQ, Self Reporting Questionnaire; STAI, State-Trait Anxiety Inventory.

We found that interventions delivered during pregnancy and postnatally had a significant overall effect compared to usual care (*n* = 15,816; ES −0.26; 95% CI −0.42, −0.10), whereas those delivered only during pregnancy did not (*n* = 2,555; ES −0.46 95% −0.94, 0.01) (Figure 6). Only one trial evaluated an intervention restricted to the postnatal period [55]. Intervention timing was not associated with ES in a meta-regression analysis (β 0.16; 95% CI −0.16, 0.49).

## Discussion

Our results show there is promise for psychosocial interventions delivered by non-mental health specialists for PCMDs in middle-income countries, and corroborate findings from a previous meta-analysis [15]. We identified a group of trials distinct from this previous meta-analysis through exclusion of trials that did not meet our inclusion criteria [32],[60]–[63], exclusion of a pilot study [64] of a trial that we included [52], and inclusion of recent [50],[53] and additional [51],[54] trials. In both meta-analyses, the lack of trials from low-income countries is striking, and research to determine the feasibility and effectiveness of delivering such interventions in these countries is urgently needed.

### Study Limitations

Our findings are exploratory and should be interpreted with caution for several reasons. First, only ten trials were included, some of which were associated with an unclear risk of bias. The small number of trials made it difficult to assess small study effects, which, if present, may have led to overestimation of the true effect of interventions. Statistical analysis in three trials included in the meta-analysis did not take account of clustering [47],[49],[53]. The exclusion of these trials in the sensitivity analysis that included only trials at low risk of bias reduced the overall ES for PCMD symptoms and caseness, suggesting that trials that did not take account of clustering may have received more weight in the meta-analysis than is appropriate. Second, interventions differed in terms of participants, timing, setting, personnel, duration, and delivery mode, and meta-analyses showed high levels of statistical heterogeneity. However, the overall impact of psychosocial interventions on PCMDs was clear, and heterogeneity was reduced in subgroup analyses of psychological and health promotion interventions. Third, we excluded trials reported in all languages other than English, French, or Spanish. Fourth, the comparison group in most trials was usual perinatal care, which, in many settings, is likely to amount to no care. Beneficial effects of interventions in these trials are therefore not surprising, and future trials should consider more active comparison groups to control for nonspecific effects of contact with health workers and for ethical reasons. Finally, trials included in the meta-analysis were all from middle-income countries, and most were from Asia. The generalizability of the study findings for low-income and non-Asian countries is therefore limited.

### Addressing PCMDs through Psychological Intervention

Our results suggest that psychological interventions for PCMDs are effective. Because we identified only three trials of psychological interventions, it is not possible to recommend one form of psychological therapy over another. However, meta-analyses combining trials from high-income countries and low- and middle-income countries have shown that CBT-based interventions are effective in reducing levels of PCMDs [7],[9]. Moreover, a meta-analysis of psychological interventions for general adult depression and anxiety disorders in low- and middle-income countries found that CBT-based interventions had significantly larger ESs than interventions incorporating other therapies [65]. IPT-based interventions have also shown promise in resource-constrained settings: two trials in rural Uganda showed strong benefits of group IPT interventions delivered by non-mental health specialists for treating general depression in adults and adolescents [66],[67].

Despite these apparent benefits of psychological interventions for common mental disorders, the interventions must be adapted for individual contexts [68]. For example, where strong gender inequalities exist, it may be unrealistic to expect a psychological intervention to empower women in a way that they are individually able to negotiate for change in their lives. Also, there may be stigma associated with participation in an intervention explicitly for mental illness. The Thinking Healthy CBT intervention for depressed Pakistani mothers addressed these contextual factors by using infant health to mobilize family members to improve conditions for the infant's mother, and by integrating the intervention into an existing community health program [56].

We included one psychological intervention to treat participants with established PCMDs and two to prevent PCMDs. Psychological interventions may be more human-resource intensive than other interventions, since they require qualified trainers and supervisors, as well as multiple sessions to build rapport between the participant and “therapist.” Delivering preventive psychological interventions to all pregnant women or new mothers is unlikely to be cost-effective, particularly in remote rural contexts without access to mental health care. Further data on the sustainability and affordability of these programs is therefore required.

### Health Promotion Interventions: Addressing Determinants of PCMDs

Although psychological interventions were associated with a significantly larger ES, we found that health promotion interventions also reduced symptoms of PCMDs compared to usual care. Health promotion interventions were diverse but had two common components: sharing information and developing skills to enhance perinatal health—though not specifically perinatal *mental health*—and giving women an opportunity to share concerns and feelings and receive social support in the context of a group or individually. Tripathy et al. hypothesized that their participatory intervention with women's groups also developed problem-solving skills and empowered women in their communities; this may account for the positive result reported in this trial in its final year [40]. Evidence from qualitative studies suggests that women with common mental disorders do not consider themselves to be ill but attribute their symptoms to social difficulties [69]–[71]. More social and less individual-focused interventions involving health promotion approaches may therefore be more acceptable.

Although health promotion interventions did not directly address mental illness, they did address determinants of PCMDs, such as poor maternal health, infant mortality, and lack of social support. Numerous general community-based interventions in low- and middle-income countries have successfully addressed risk factors for PCMDs, for example, domestic violence [72], poor access to maternal health care [73], and neonatal mortality [74],[75]. More explicit recognition of women's mental health as both a mediator and consequence of these outcomes may increase the effectiveness of such interventions (both in terms of improving women's mental health as well as other targets including reduction in domestic violence), and future trials should consider incorporating a mental health outcome.

We were unable to carry out a subgroup analysis of treatment versus preventive interventions because only one treatment intervention was identified [56]. However, all seven health promotion interventions adopted a preventive approach. In the context of low- and middle-income countries, preventive interventions have several advantages over treatment interventions. First, the chance of detecting PCMDs in low- and middle-income countries is low if access to health care is low in the perinatal period. An intervention involving assessment of mental health status prior to participation may therefore be unrealistic. Preventive interventions are not necessarily dependent on detection of mental illness. Second, some of the effects of PCMDs on infants are thought to begin within the first few months after birth [76]. Delayed diagnosis and treatment could therefore lead to early disruption of the mother–infant relationship, as well as an extended period of distress for the mother. A preventive intervention might avoid these harmful effects. Third, training and supervising personnel to deliver psychological or pharmacological treatment interventions may be more laborious and costly than training personnel to deliver preventive interventions addressing social determinants of PCMDs. Finally, preventive interventions that reduce population levels of domestic violence, poverty, and reproductive ill health that perpetuate mental illness are likely to have a long-term impact on the prevalence of PCMDs.

### Delivery of Psychosocial Interventions

Although delivery method was not associated with ES, we found evidence that interventions delivered through groups reduced symptoms of PCMDs compared to usual care, and that group-based interventions were associated with a larger ES than individual interventions. The “one-to-many” approach employed by group interventions is attractive in resource-constrained settings and where it is more culturally appropriate for women to come together to discuss their problems rather than having one-to-one discussions with a health professional. Previous meta-analyses that included subgroup analyses of group interventions for PCMDs in high-income countries reported inconsistent results: one study reported no overall reduction in postnatal depressive symptoms; another study showed a significant effect of group interventions compared to individual interventions [7],[8]. Such contradictory results have led some authors to question the efficacy of psychological group interventions for mothers with young children [77]. In contrast, three meta-analyses of trials from high-income countries all reported that individual interventions reduced levels of PCMD compared to usual care [7]–[9]. The fact that interventions incorporating both group and individual components did not have an impact on PCMDs warrants further investigation. However, only two trials were included in this subgroup, and the finding should therefore be interpreted with caution.

### Onset, Duration, and Intensity of Intervention

We found that interventions delivered during pregnancy and postnatally were associated with a reduction in symptoms of PCMDs compared to usual care. The fact that interventions restricted to pregnancy had no significant effect on PCMDs compared to usual care suggests that intervention in the postnatal period is important. In support of this, a meta-analysis of trials from high-income countries showed that psychosocial interventions delivered postnatally prevented postnatal depression compared to usual care, whereas those beginning antenatally and continuing postnatally had no effect [8]. Postnatal psychosocial interventions may be more beneficial because women rely on social support and emotional resilience in the postnatal period to care for a newborn, recover from childbirth, and resume their daily routines. Interventions addressing anxieties around childbirth and perinatal health may be more appropriate for pregnant women.

In the current review the duration and intensity of interventions was variable but did not appear to be correlated with ES. There is little evidence in the literature for an optimum, or even minimum, number or frequency of sessions, although findings from a meta-analysis of trials in high-income countries indicated that interventions involving a single contact event do not prevent postnatal depression, whereas interventions with multiple contact events are efficacious [8].

### Personnel

A recent review of possible packages of care for depression in low- and middle-income countries included routine screening for detection of depression, psychoeducation, and problem-solving [78]. This meta-analysis and other key trials of interventions for general common mental disorders provide some evidence that community health workers or lay workers can deliver these non-pharmacological interventions [67],[79]–[81]. Advantages of working with these cadres are that interventions can be delivered at the community level and in areas without access to mental health care. However, community health workers are already indispensable in the provision of perinatal care, family planning, health education, HIV/AIDs care, and immunization programs. Their existing workload may limit their availability for mental health interventions. Referral of severe mental illness must also be considered, and nesting interventions in existing health care services where specialist care and pharmacotherapy can be provided is one potential strategy. Trials of interventions integrated into primary care settings in India and Chile have reported promising results [32],[81],[82]. Further consideration is needed to adapt existing mental health care packages for PCMDs. For example, routine screening for PCMDs has been demonstrated to be not cost-effective in high-income countries, and could overwhelm weak health systems in low- and middle-income countries [83].

### Conclusions

Evidence supports the implementation of psychosocial interventions for PCMDs delivered by non-mental health specialists in middle-income countries. We found stronger evidence for the efficacy of psychological interventions, compared to health promotion interventions. More research is needed to evaluate the impact of such interventions in low-income countries, as well as research to compare treatment and preventive approaches, and antenatal versus postnatal interventions.

## Supporting Information

Figure S1 **Assessment of small study effects on ES of psychosocial interventions for PCMDs.**
(TIF)

Protocol S1 **Protocol for a systematic review and meta-analysis of psychosocial interventions for PCMDs delivered by non-mental health specialists in low- and middle-income countries.**
(DOC)

Table S1 **Trials excluded at the final stage of screening and reasons for exclusion.**
(DOC)

Table S2 **Ongoing trials of psychosocial interventions for PCMDs delivered by non-mental health specialists in low- and middle-income countries.**
(DOC)

Table S3 **Meta-regression analyses of potential predictors of the effect of psychosocial interventions for PCMDs.**
(DOC)

Text S1 **PRISMA checklist of items to be reported in a systematic review or meta-analysis.**
(DOC)

Text S2 **Search terms for Embase via OVID.**
(DOC)

## Abbreviations

CBT: cognitive behavior therapy
ES: effect size
IPT: interpersonal therapy
OR: odds ratio
PCMD: perinatal common mental disorder
