# Correction: Psychosocial Interventions for Perinatal Common Mental Disorders Delivered by Providers Who Are Not Mental Health Specialists in Low- and Middle-Income Countries: A Systematic Review and Meta-Analysis

**DOI:** 10.1371/journal.pmed.1001678

**Published:** 2014-06-25

**Authors:** The PLOS Medicine Staff

## Abstract

*Please see later in the article for the Editors' Summary*

In the article Clarke K, King M, Prost A (2013) Psychosocial Interventions for Perinatal Common Mental Disorders Delivered by Providers Who Are Not Mental Health Specialists in Low- and Middle-Income Countries: A Systematic Review and Meta-Analysis. PLoS Med 10(10): e1001541. doi:10.1371/journal.pmed.1001541, the authors discovered that an analysis error occurred when they changed statistical programs as part of a revision, and resulted in pooling odds ratios rather than log odds ratios. This analysis error led to several errors in the manuscript, as follows:

The fourth sentence of the Abstract Methods and Findings should read: “Interventions led to an overall reduction in PCMDs compared to usual care when using continuous data for PCMD symptomatology (effect size [ES] -0.34; 95% CI -0.53, -0.16) but not binary categorizations for presence or absence of PCMDs (OR 0.62, 95% CI 0.35, 1.08).”

In Methods, Data synthesis and statistical analysis, the fifth sentence should read: “Natural log Odds Ratios (ORs) were pooled for trials reporting binary outcomes.”

In Results, Comparison 1: All Interventions versus Usual Care, the second and third sentences in the first paragraph should read: “There was evidence that interventions delivered by non-mental health specialists compared to usual perinatal care were associated with a reduction in PCMD symptoms (ES -0.34; 95% CI -0.53, -0.16), but not caseness (OR 0.62, 95% CI 0.35, 1.08), immediately after the intervention. Heterogeneity was high (I2  =  83.9% and 80.2%, respectively) and statistically significant.” The second paragraph of this section should be replaced by: “These analyses resulted in similar ESs for continuous outcomes. For binary outcomes, excluding the non-peered reviewed study (OR 0.55; 95% 0.31, 0.96) and pooling outcomes associated with the final assessment (OR 0.56, 95% CI 0.33, 0.95) were associated with significant effects. We also performed a sensitivity analysis using studies with low risk of bias and found that the ES was reduced for PCMD symptoms and caseness (ES -0.19; 95% CI -0.36, -0.02; OR 0.65; 95% CI 0.34, 1.23).”

The following sentence should be inserted as the first sentence of Discussion Study Limitations section: “Our conclusions are limited by the inconsistency between results for continuous and binary outcomes, though analysis using the latter results in a loss of information and is therefore less sensitive.” The seventh sentence of the section should read: “However, heterogeneity was reduced in subgroup analyses of psychological and health promotion interventions.”

Figure 3. The Figure shown should replace Figure 3. The revised pooled result is no longer significant at OR 0.62; 95% CI 0.35, 1.08. Pooling ORs slightly affected the weighting of individual studies. Heterogeneity remained significant at p<0.001. The first sentence of the legend of Figure 3 should read: “Using binary PCMD categorizations from assessments immediately following delivery of the intervention, the pooled effect for all interventions was not significant (OR 0.62; 95% CI 0.35, 1.08) compared to usual care.”

**Figure 3:**
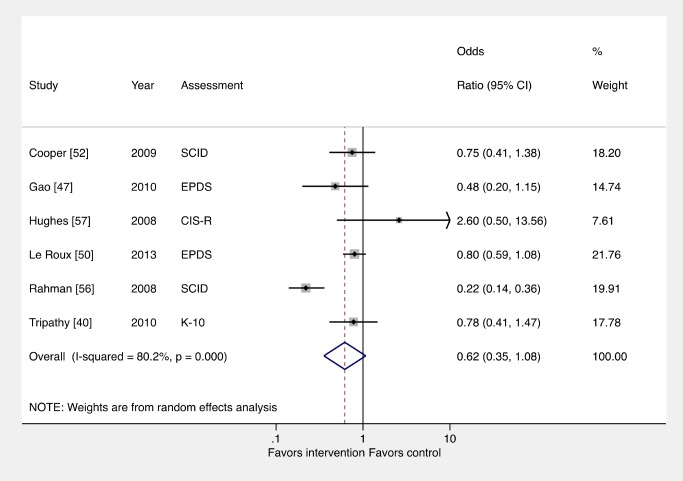
Effects of psychosocial interventions on binary PCMD outcomes. Using binary PCMD categorizations from assessments immediately following delivery of the intervention, the pooled effect for all interventions was not significant (OR 0.62; 95% CI 0.35, 1.08) compared to usual care. CIS-R, Clinical Interview Schedule–Revised; EPDS, Edinburgh Postnatal Depression Scale; K-10, Kessler 10-Item Scale; SCID, Structured Clinical Interview for DSM Disorders.

Although the corrected result pooling binary outcomes was not significant, the authors’ main conclusion, that there is evidence that psychosocial interventions delivered by non-specialists are beneficial for PCMDs, continues to be supported by the pooled effect size based on continuous outcomes (Figure 2), which remains significant. Furthermore, the effect size pooling binary and continuous outcomes using converted ORs also remains significant (Figure 4: ES -0.27; 95% CI -0.42, -0.13).

In the Editors’ Summary, What Did the Researchers Do and Find?, the fourth sentence should read: “Combining results from the ten remaining studies, the researchers found that compared to usual perinatal care (which in most cases included no mental health care), interventions delivered by providers who were not mental health specialists were associated with an overall reduction in mental health symptoms, but were not associated with a decreased likelihood of being diagnosed with a mental health disorder”.
