# Supplementary figures and images for: Psychosocial Interventions for Perinatal Common Mental Disorders Delivered by Providers Who Are Not Mental Health Specialists in Low- and Middle-Income Countries: A Systematic Review and Meta-Analysis
Source: PLoS Med. 2013 Oct 29;10(10):e1001541. doi: 10.1371/journal.pmed.1001541 (PMC3812075; doi:10.1371/journal.pmed.1001541)

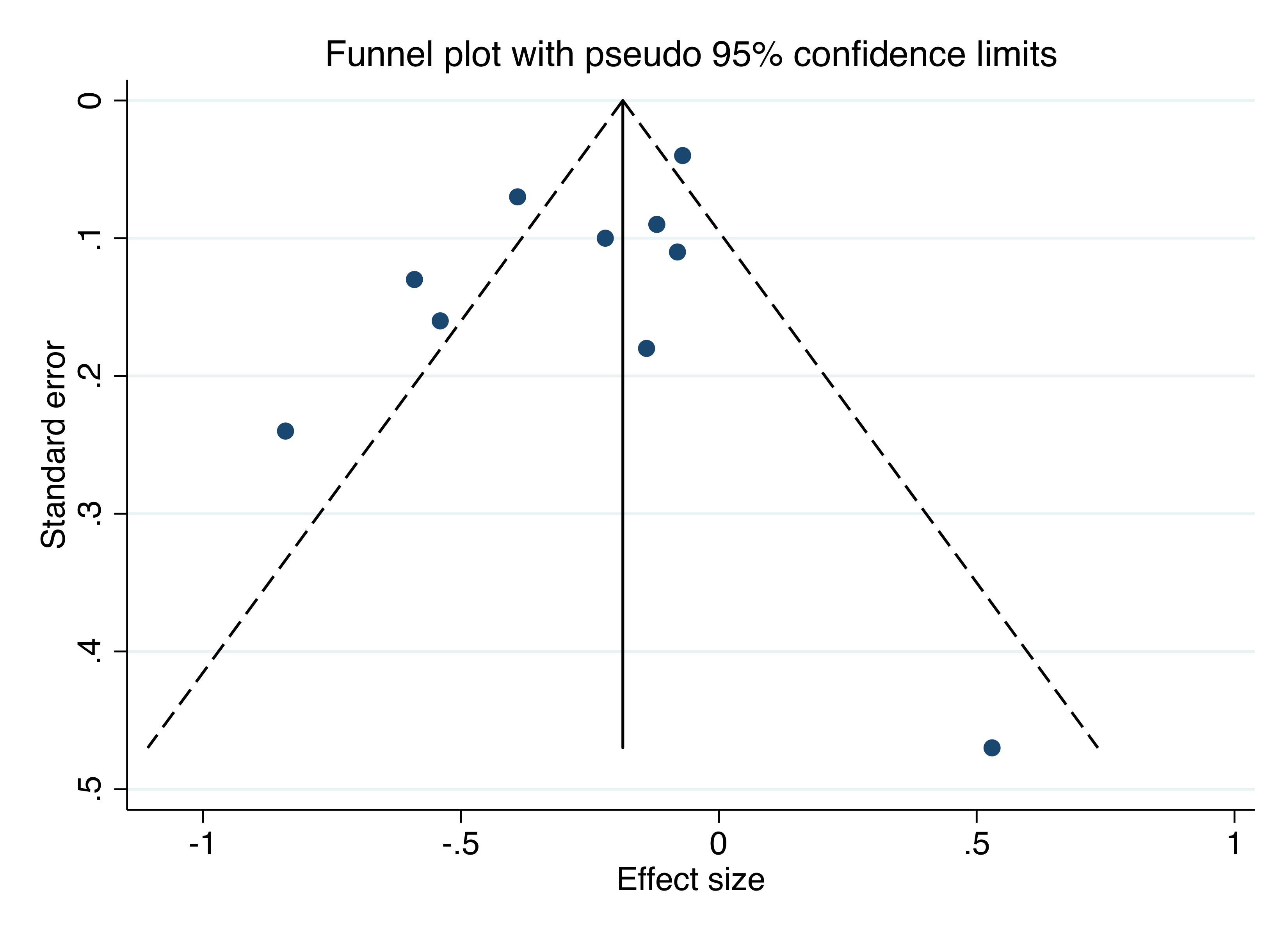

Supplement: Figure S1 — Assessment of small study effects on ES of psychosocial interventions for PCMDs. (TIF) [file pmed.1001541.s001.tif]
