# Supplementary material for: Psychosocial Interventions for Perinatal Common Mental Disorders Delivered by Providers Who Are Not Mental Health Specialists in Low- and Middle-Income Countries: A Systematic Review and Meta-Analysis
Source: PLoS Med. 2013 Oct 29;10(10):e1001541. doi: 10.1371/journal.pmed.1001541 (PMC3812075; doi:10.1371/journal.pmed.1001541)
Supplement: Protocol S1 — Protocol for a systematic review and meta-analysis of psychosocial interventions for PCMDs delivered by non-mental health specialists in low- and middle-income countries. (DOC) [file pmed.1001541.s002.doc]

**Protocol S1: Protocol for a systematic review and meta-analysis of psychosocial interventions for perinatal common mental disorders delivered by non-mental health specialists in low- and middle-income countries**

**Review questions**

1. What psychosocial interventions for PCMDs, delivered by non-mental health specialists, have been implemented in low- and middle-income countries?

2. What is the overall effect of these psychosocial interventions on PCMD outcomes versus usual perinatal care?

3. What are the key components of effective psychosocial interventions with regards to intervention type, content, delivery mode, personnel, duration, timing, intensity, and target disorder?

**Search strategy**

We will search the following databases: Medline, Embase, Cumulative Index to Nursing and Allied Health Literature, the Cochrane Library, PsycINFO, Web of Science, Scopus, Popline, Maternity and Infant Care, the Global Health Library and The World Health Organization International Clinical Trials Registry. Customized search strategies will be developed for each database using the keywords in Table 1. We will use controlled vocabulary (e.g. MeSH terms) and search filters where available, otherwise Boolean search methods and free text terms will be employed.

| PCMD | Perinatal |
| --- | --- |
| common mental disorders  anxiety  depression  distress  panic disorder  depressive disorder  depressive symptoms  anxious symptoms  somatization symptoms  somatisation symptoms  somatization disorder  somatisation disorder  somatoform symptoms | maternal  antenatal  perinatal  pregnancy  postpartum  postnatal  puerperal |

Table 1: Key words used in literature search strategy^a^

Note: search limited to studies conducted in World Bank defined low- and middle-income countries.

**Trial selection criteria**

**Inclusion criteria**

- Psychosocial interventions that aim to influence thoughts, behaviors, skills and/or associated feelings
- Conducted in low-and middle-income countries using World Bank country classifications
- Trial design: randomized or non-randomized controlled trials
- Article written in English, Spanish or French
- Participants: women with or without a diagnosed PCMD, categorized as follows: (i) pregnant on recruitment; (ii) postnatal on recruitment (up to 12 months postpartum); (iii) not pregnant on recruitment but became pregnant during the course of the trial
- Interventions: psychosocial interventions to prevent or treat PCMDs delivered prior to pregnancy, during pregnancy or during the postpartum, delivered by non-mental health specialists (i.e. lay persons, health workers, health volunteers, nurses and primary care doctors).
- Setting: community (e.g. villages), or the most commonly accessible health provider of antenatal care in the location (e.g. health posts, primary care centers and hospitals).
- PCMD outcomes: caseness or symptoms of depression, anxiety, panic and somatic disorder during pregnancy or up to 12 months postpartum. Also perinatal psychological distress as a proxy measure of PCMDs.
- Assessment of PCMDs: structured clinical interviews or a screening questionnaire validated in the same country in which the trial was conducted.
- Publication dates will not be restricted

**Exclusion criteria**

- Interventions delivered by mental health specialists, such as psychiatrists, psychologists and psychosocial workers, as these practitioners are not commonly available in low-and middle-income country contexts.
- Trials assessing a PCMD outcome after the first 12 months postpartum.
- Trials using a non-validated screening assessment.

**Data extraction**

KC will screen all studies and assess for bias. KC and AP will extract study data. Any confusion regarding the inclusion or exclusion of studies will be resolved through discussions between all three authors (KC, AP, MK).

We will extract data for the following fields:

- Date of extraction
- Source reference and type
- Author
- Year of publication/registration
- Article title
- Source of funding
- Trial design
- Study setting (i.e. country, facility/community setting)
- Target disorder
- Description of intervention (i.e. content, delivery mode)
- Timing and duration of intervention
- Description of care received by control group
- Personnel (e.g. community health worker, lay person)
- Participant selection criteria
- Participant exclusion criteria
- Number of subjects eligible for trial
- Number of subjects allocated to intervention or control arm
- Number of subjects lost to follow up
- Number of participants in each arm of the trial
- Assessment tool used and if it was validated
- Details of timing of assessments
- Definitions of caseness (i.e. cut off point) if a continuous outcome is dichotomized
- Results at assessment immediately after delivery of the intervention (e.g. relative risk or odds ratio for dichotomous outcomes, and mean difference for continuous outcomes, plus confidence intervals)
- Results at the final assessment
- Variables adjusted for in trial analyses.

**Assessment of risk of bias**

KC will use the Cochrane Risk of Bias Tool to judge risk of bias across the following domains: sequence generation, allocation concealment, blinding of participants and personnel, blinding of outcome assessors, incomplete outcome data and selective reporting [1]. If ten or more studies are identified we will assess potential publication bias and small study effects with funnel plots and Egger tests.

**Data synthesis plan**

A meta-analysis will be conducted if at least six studies that are not at high risk of bias are identified, and interventions and samples are comparable [2]. We will not exclude trials from the systematic review on the basis of methodological quality, in order to identify all psychosocial interventions delivered by non-mental health specialists in low- and middle-income countries. However, trials at high risk of bias – i.e. those that are found to be at high risk or unclear risk of bias across five or more bias domains - will be excluded from the meta-analysis. This is because results from these trials may be invalid and produce misleading results in the meta-analysis, although admittedly this exclusion threshold is arbitrary, and in reality studies may lie anywhere along the continuum from ‘free of bias’ to ‘undoubtedly biased’ [3]. We will conduct a sensitivity analysis including studies at low risk of bias only – ‘low risk’ trials will be defined as those using adequate sequence generation and allocation concealment methods.

If a meta-analysis is conducted the following comparisons will be made:

1. All interventions versus usual care

2. Treatment interventions versus usual care

3. Preventive interventions versus usual care

We aim to conduct subgroup analyses to investigate effects of psychological and other intervention types, and group and individually delivered interventions versus usual care. Meta-analyses will use results from assessments carried out immediately after the intervention; sensitivity analyses will therefore be conducted to assess the efficacy of interventions using results from the final assessment of PCMD.

**References**

1. Higgins JPT, Altman DG, Gøtzsche PC, Jüni P, Moher D, et al. (2011) The Cochrane Collaboration's tool for assessing risk of bias in randomised trials. British Medical Journal 343: d5828.

2. Fu R, Gartlehner G, Grant M, Shamilyan T, Sedrakyan A, et al. (2011) Conducting quantitative synthesis when comparing medical interventions: AHRQ and the Effective Health Care Program. Journal of Clinical Epidemiology 64: 1187-1197.

3. Higgins JPT, Green S (2011) Cochrane Handbook for Systematic Reviews of Interventions Version 5.1.0 [updated March 2011]
