# Supplementary material for: Psychosocial Interventions for Perinatal Common Mental Disorders Delivered by Providers Who Are Not Mental Health Specialists in Low- and Middle-Income Countries: A Systematic Review and Meta-Analysis
Source: PLoS Med. 2013 Oct 29;10(10):e1001541. doi: 10.1371/journal.pmed.1001541 (PMC3812075; doi:10.1371/journal.pmed.1001541)
Supplement: Table S1 — Trials excluded at the final stage of screening and reasons for exclusion. (DOC) [file pmed.1001541.s003.doc]

**Table S1: Trials excluded at the final stage of screening and reasons for exclusion**

| Study | Reason for exclusion |
| --- | --- |
| Ali 2010 [1] | Involved an inappropriate control group consisting of women who refused the intervention but agreed to screening. |
| Baker-Henningham 2005 [2] | PCMD outcome was assessed beyond 12 months postpartum. |
| Ciftçi 2012 [3] | Insufficient information was provided to identify who delivered the intervention and the authors failed to respond to an email request for this information. |
| Jareethum 2008 [4] | Insufficient information was provided about the tool used to measure anxiety |
| Kalinauskiene 2009 [5] | Maternal depression was not an outcome in this trial. |
| Langer 1998 [6] | The intervention was delivered at a labor and delivery unit in a hospital, i.e. not the most commonly accessible facility for antenatal care. |
| Lara 2010 [7] | The intervention was delivered by psychologists, i.e. mental health specialists. |
| Mundell 2011 [8] | Masters level psychology students facilitated the intervention. |
| Miquelutti 2012 [9] | Only a conference abstract was identified and therefore insufficient information was available to assess the intervention and trial design. The authors failed to respond to an email request for further information. |
| Morris 2012 [10] | The PCMD outcome was assessed beyond 12 months postpartum for a significant proportion of participants. |
| Ozer 2011 [11] | PCMD outcome was assessed beyond 12 months postpartum. |
| Wolman 1993 [12] | The intervention was delivered in the labor room of a hospital, i.e. not the most commonly accessible health provider of antenatal care. |
| Tezel 2006 | No control group |
| Husain 2011 [13] | The trial is ongoing and the intervention is being delivered at a teaching hospital, i.e. not the most commonly accessible facility for antenatal care. |
| Jahdi 2012 [14] | The trial is ongoing and the intervention is being delivered at a hospital that does not commonly provide antenatal care. |
| Shariat 2013 [15] | The trial is ongoing and the intervention is being delivered at a hospital that does not commonly provide antenatal care. |

References

1. Ali NS, Ali BS, Azam IS, Khuwaja AK (2010) Effectiveness of counseling for anxiety and depression in mothers of children ages 0-30 months by community workers in Karachi, Pakistan: a quasi experimental study. BMC Psychiatry 10: 57.

2. Baker-Henningham H, Powell C, Walker S, Grantham-McGregor S (2005) The effect of early stimulation on maternal depression: a cluster randomised controlled trial. Archives of Disease in Childhood 90: 1230-1234.

3. Ciftçi EK, Arikan D (2012) The effect of training administered to working mothers on maternal anxiety levels and breastfeeding habits. Journal of Clinical Nursing 21: 2170-2178.

4. Jareethum R, Titapant V, Chantra T, Sommai V, Chuenwattana P, et al. (2008) Satisfaction of healthy pregnant women receiving short message service via mobile phone for prenatal support: A randomized controlled trial. Journal of the Medical Association of Thailand 91: 458-463.

5. Kalinauskiene L, Cekuoliene D, Ijzendoorn MH, Bakermans-Kranenburg MJ, Juffer F, et al. (2009) Supporting insensitive mothers: the Vilnius randomized control trial of video-feedback intervention to promote maternal sensitivity and infant attachment security. Child: Care, Health and Development 35: 613-623.

6. Langer A, Campero L, Garcia C, Reynoso S (1998) Effects of psychosocial support during labour and childbirth on breastfeeding, medical interventions, and mothers' wellbeing in a Mexican public hospital: a randomised clinical trial. British Journal of Obstetrics and Gynaecology. pp. 1056-1063.

7. Lara MA, Navarro C, Navarrete L (2010) Outcome results of a psycho-educational intervention in pregnancy to prevent PPD: a randomized control trial. Journal of Affective Disorders 122: 109-117.

8. Mundell JP, Visser MJ, Makin JD, Kershaw TS, Forsyth BW, et al. (2011) The impact of structured support groups for pregnant South African women recently diagnosed HIV positive. Women & Health 51: 546-565.

9. Miquelutti MA, Cecatti JG, Makuch MY. Evaluation of the efficacy of an antenatal birth preparation program. In: International Journal of Gynecology and Obstetrics, editor; 2012 October; Rome. pp. S414.

10. Morris J, Jones L, Berrino A, Jordans MJD, Okema L, et al. (2012) Does combining infant stimulation with emergency feeding improve psychosocial outcomes for displaced mothers and babies? A controlled evaluation from northern Uganda. American Journal of Orthopsychiatry 82: 349-357.

11. Ozer EJ, Fernald LCH, Weber A, Flynn EP, VanderWeele TJ (2011) Does alleviating poverty affect mothers' depressive symptoms? A quasi-experimental investigation of Mexico's Oportunidades programme. International Journal of Epidemiology 40: 1565-1576.

12. Wolman W, Chalmers B, Hofmeyr GJ, Nikodem VC (1993) Postpartum depression and companionship in the clinical birth environment: a randomized, controlled study. American Journal of Obstetrics & Gynecology 168: 1388-1393.

13. Husain N (2011) Trial for postnatal depression. In: US National Insititutes of Health, editor. ClinicalTrialsgov,.

14. Jahdi F (2012) The effect of a prenatal yoga programme in the second and third trimester of pregnancy on the anxiety and pregnancy outcomes at primiparouse[sic] women. In: Iranian Registry of Clinical Trials, editor.

15. Shariat M (2013) Maternal mood disorder and its effects on infant sleep and effect of behavioral and educational interventions on maternal and infant sleep. In: Iranian Registry of Clinical Trials, editor.
