# Supplementary material for: Psychosocial Interventions for Perinatal Common Mental Disorders Delivered by Providers Who Are Not Mental Health Specialists in Low- and Middle-Income Countries: A Systematic Review and Meta-Analysis
Source: PLoS Med. 2013 Oct 29;10(10):e1001541. doi: 10.1371/journal.pmed.1001541 (PMC3812075; doi:10.1371/journal.pmed.1001541)
Supplement: Table S2 — Ongoing trials of psychosocial interventions for PCMDs delivered by non-mental health specialists in low- and middle-income countries. (DOC) [file pmed.1001541.s004.doc]

**Table S2: Ongoing trials of psychosocial interventions for perinatal common mental disorders delivered by non-mental health specialists in low and middle-income countries**

|  | Setting | Details |
| --- | --- | --- |
| South Asian Hub for Advocacy, Research and Education on mental health (SHARE) [1] | Goa, India  Rawalpindi, Pakistan | Implementation of an adapted Thinking Healthy Program, to be delivered by peers (not Lady Community Health Workers), to reduce maternal depression. |
| Program for Improving Mental Health Care (PRIME) [2] | Selected districts or sub-districts in: Ethiopia, India,  Nepal, South Africa, Uganda | Integration of mental health, including maternal mental health, into primary care through country-specific research activities |
| Project Masihambisane [3] | Rural areas in Kwa-Zulu-Natal, South Africa | Trial of an intervention with peer mentors comprising four antenatal and four postnatal group sessions for women living with HIV. Sessions cover various issues related to perinatal and HIV-related health. The EPDS and GHQ are being used to collect maternal mental health data. |
| Community-based package for maternal, newborn, child and HIV care [4] | Peri-urban settlement in Kwa-Zulu-Natal, South Africa | An integrated community-based package for maternal and child health and prevention against mother to child transmission of HIV, delivered through home visits by community health workers targeting pregnant and postnatal women and their newborns. Maternal depression is a secondary outcome assessed at 12 weeks postpartum. |
| The *Dil Mil* intervention [5] | Public primary health centers in Bengalaru, India | Group sessions for pregnant women with a history of domestic violence, and their mother in-laws. Sessions with pregnant women involve a participatory learning and action approach to address violent relationships and increase social support. Sessions with mother in-laws involve reflection on direct and indirect experiences of domestic violence, and the impact of these experiences on their personal health and the health of their daughter in-laws. |
| Participatory women’s groups [6,7] | Rural communities in Dhanusha district, Nepal, and Moulvibazar, Faridpur and Bogra districts in Bangladesh | Women’s groups facilitated by female community health volunteers. Group members work through participatory learning and action cycles to improve maternal and infant health and nutrition at the community level. Psychological distress is measured at six weeks postpartum using the GHQ. |

Notes: HIV Human immunodeficiency virus; EPDS Edinburgh Postnatal Depression Scale; GHQ General Health Questionnaire.

References

1. Patel V, De Silva M, Fuhr D, Weiss H (2013) SHARE: an intervention delivered by peer counsellors for maternal depression. London: Centre for Global Mental Health,.

2. Lund C, Tomlinson M, De Silva M, Fekadu A, Shidhaye R, et al. (2012) PRIME: A programme to reduce the treatment gap for mental disorders in five low-and middle-income countries. PLoS Medicine 9: e1001359.

3. Rotheram-Borus MJ, Richter L, Van Rooyen H, van Heerden A, Tomlinson M, et al. (2011) Project Masihambisane: a cluster randomised controlled trial with peer mentors to improve outcomes for pregnant mothers living with HIV. Trials 12.

4. Tomlinson M, Doherty T, Jackson D, Lawn JE, Colvin M, et al. (2011) An effectiveness study of an integrated, community-based package for maternal, newborn, child and HIV care in South Africa: study protocol for a randomized controlled trial. Trials 12.

5. Krishnan S, Subbiah K, Chandra P, Srinivasan K (2012) Minimizing risks and monitoring safety of an antenatal care intervention to mitigate domestic violence among young Indian women: The Dil Mil trial. BMC Public Health 12.

6. Clarke K (2013) Tension in the Terai: exploring maternal mental health in the perinatal period and interventions to improve it in Dhanusha district, Nepal. London: UCL.

7. Houweling TAJ, Azad K, Younes L, Kuddus A, Shaha S, et al. (2011) The effect of participatory women's groups on birth outcomes in Bangladesh: does coverage matter? Study protocol for a randomized controlled trial. Trials 12.
