# Supplementary material for: Psychosocial Interventions for Perinatal Common Mental Disorders Delivered by Providers Who Are Not Mental Health Specialists in Low- and Middle-Income Countries: A Systematic Review and Meta-Analysis
Source: PLoS Med. 2013 Oct 29;10(10):e1001541. doi: 10.1371/journal.pmed.1001541 (PMC3812075; doi:10.1371/journal.pmed.1001541)
Supplement: Table S3 — Meta-regression analyses of potential predictors of the effect of psychosocial interventions for PCMDs. (DOC) [file pmed.1001541.s005.doc]

**Table S3: Meta-regression analyses of potential predictors of the effect of psychosocial interventions for PCMDs**

| Potential predictor (covariate) | Meta-regression coefficient | P-value | Lower 95% | Upper 95% | Tau-squared^a^ | I-squared  residual^b^ | Adjusted R-squared^c^ |
| --- | --- | --- | --- | --- | --- | --- | --- |
| Intervention type  (psychological or health promotion) | -0.33 | 0.015 | -0.58 | -0.09 | <0.00 | 48.8% | 91.8% |
| Delivery method  (group, individual or group and individually delivered) | -0.11 | 0.355 | -0.36 | 0.14 | 0.04 | 78.7% | -8.4% |
| Timing  (antenatal, postnatal, antenatal and postnatal) | 0.16 | 0.275 | -0.16 | 0.49 | 0.05 | 80.6% | -13.4% |

^a^ Remaining between-study variance in the outcome variable not explained by the covariate

^b^ Proportion of residual between-study variation in the outcome variable attributable to heterogeneity, not sampling variability

^c^ Relative reduction in between study variance of the outcome variable due to inclusion of the covariate in the model
